# Using Free Internet Videogames in Upper Extremity Motor Training for Children with Cerebral Palsy

**DOI:** 10.3390/bs6020010

**Published:** 2016-06-07

**Authors:** Marisa Sevick, Elizabeth Eklund, Allison Mensch, Matthew Foreman, John Standeven, Jack Engsberg

**Affiliations:** 1 Creighton Pediatric Therapy, 17055 Frances Street, Suite 103, Omaha, NE 68130, USA; MarisaSevick@creighton.edu; 2 School of Medicine, Washington University in St. Louis, St. Louis, MO 63108, USA; eklunde88@gmail.com (E.E.); allisonmensch@gmail.com (A.M.); foremanm@wustl.edu (M.F.); standeve@netscape.net (J.S.)

**Keywords:** feasibility, motivation, Internet videogames, Kinect, FAAST software, cerebral palsy

## Abstract

Movement therapy is one type of upper extremity intervention for children with cerebral palsy (CP) to improve function. It requires high-intensity, repetitive and task-specific training. Tedium and lack of motivation are substantial barriers to completing the training. An approach to overcome these barriers is to couple the movement therapy with videogames. This investigation: (1) tested the feasibility of delivering a free Internet videogame upper extremity motor intervention to four children with CP (aged 8–17 years) with mild to moderate limitations to upper limb function; and (2) determined the level of intrinsic motivation during the intervention. The intervention used free Internet videogames in conjunction with the Microsoft Kinect motion sensor and the Flexible Action and Articulated Skeleton Toolkit software (FAAST) software. Results indicated that the intervention could be successfully delivered in the laboratory and the home, and pre- and post- impairment, function and performance assessments were possible. Results also indicated a high level of motivation among the participants. It was concluded that the use of inexpensive hardware and software in conjunction with free Internet videogames has the potential to be very motivating in helping to improve the upper extremity abilities of children with CP. Future work should include results from additional participants and from a control group in a randomized controlled trial to establish efficacy.

## 1. Introduction

Cerebral palsy (CP) involves a group of disorders that affects a person’s movement abilities. These disorders can disrupt the individual’s ability to control his/her muscles, movements and posture. It is estimated that three per 1000 children in the U.S. have CP [1]. Children with CP often experience difficulties related to motor control in their upper extremities (UE), including reaching, grasping and manipulation. These activities are jerkier, slower, less forceful and less direct in children with CP than typically-developing children [2]. Impairments in the UE can limit children’s functional abilities in many of their occupations.

Movement therapy is one type of UE intervention implemented for children with CP to improve their functioning [3,4]. This therapy requires high-intensity, repetitive and task-specific movement training to improve performance. Tedium and lack of motivation are substantial barriers to performance improvement [5,6,7,8]. An approach to overcome these barriers is to couple the movement therapy with virtual reality (VR) activities, which may include videogames. Research suggests that using motion-based VR for rehabilitation can provide a very engaging and motivating approach for therapy [2,7,8,9,10]. Patients who participate in VR gaming therapy increase the duration, frequency and intensity of therapy movements, leading to enhanced motor performance [4,10,11,12,13]. Despite the benefits of VR, a number of limitations can inhibit the implementation of VR-based therapy. Some systems currently used for UE training require specific devices to control the systems [2,14,15,16,17]. These devices have shown improvement in functioning for children with CP; however, some may be cumbersome, difficult to set up, may not allow for use in the home and are limited in the segments/joints they can train. In addition, games for many of these systems are written by computer scientists and engineers making the games expensive and limited in selection [4,13]. Off-the-shelf games with movement sensors (*i.e.*, Wii™) may be used, but may not specifically meet the needs of individuals who have an impairment (e.g., weakness, inadequate range of motion and poor motor control) [18].

Another approach for this type of therapy is using the vast number of videogames that are freely available via the Internet [19]. Internet games eliminate the high cost of new game development, permit games to be paired with an individual’s interests and allow for changing of games to maintain novelty. Matching the interest of the child makes the therapy increasingly client-centered and motivating. The Internet games are diverse, high quality, adequately complex for continued motivation, novel and adaptable for therapy.

Coupling the free Internet videogames with the Microsoft Kinect (~$100) movement sensor [19] and the free Flexible Action and Articulated Skeleton Toolkit software (FAAST) [20] permits almost any videogame to be used for movement therapy [21]. Therapists can quickly learn the techniques and easily create individualized movement therapy regimens for their clients [21]. The use of the sensor and software paired with videogames has not been extensively tested in persons with motor disabilities.

The purposes of this investigation were to: (1) test the feasibility of delivering our upper extremity motor training intervention to children with cerebral palsy; and (2) determine the level of intrinsic motivation during intervention participation. Results indicated that the intervention could be successfully delivered in the laboratory and the home, and pre- and post- assessments were possible. Results also indicated a high level of motivation among the participants.

## 2. Results

### 2.1. Feasibility of Intervention Delivery

In total, there were 26 different games played by the children over the course of the intervention (Table 1). Some games were played by all children, and some were selected based solely on the preferences of the individual child.

Recorded data from the Kinect and FAAST software indicated that the four participants completed all 12 weeks of the intervention and demonstrated success in using equipment and software in their homes. Due to family preferences, Participant 1 did not progress to the intervention fully taking place in the home. This participant continued coming to the laboratory two times per week and completed one session at home per week for the last nine weeks of the intervention. The remaining participants progressed through the pre-set 12-week plan.

All participants obtained a high number of repetitions during training sessions. On average, Participant 1 obtained about 500 repetitions per session. Participant 2 completed about 640 repetitions per session. Participant 3 completed an average of 850 repetitions per session. Participant 4 obtained an average of 1480 repetitions per session.

**Figure 1:**
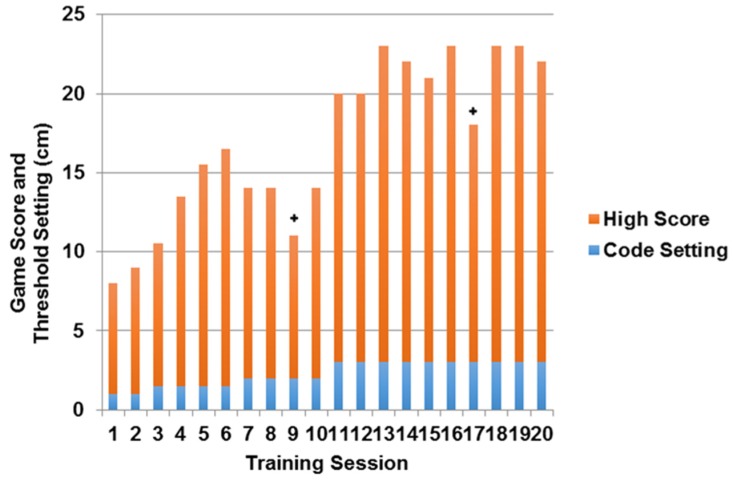
Example of high game score and game success threshold setting over the course of 20 sessions for Participant 1. + Denotes day of technical difficulty that prevented best effort.

The score of the “basketball shot” game for Participant 1 continued to increase over the first 12 training sessions (Figure 1), except in cases where technical difficulties occurred. Slight decreases in game score occurred when the “success” threshold was increased, but adaptation to the greater difficulty was quickly made (Figure 1). During the last eight sessions, there was a plateau in both game score and threshold setting.

The three different assessment types were successfully collected before and after the intervention. For the active range of motion (AROM), no meaningful changes were noted for Participants 1 and 2. Participant 3 showed an increase in AROM for shoulder flexion, abduction and external rotation in his affected UE. Participant 4 showed an increase in AROM for shoulder flexion and abduction and wrist flexion and extension in his affected UE (Table 2).

Data were successfully collected using the Bruininks–Oseretsky Test of Motor Proficiency (BOT-2) for Participants 3 and 4. There were no changes for Participant 3 (Table 3). On the other hand, Participant 4 had an increase in upper limb coordination. Participant 4 also showed an increase in manual coordination overall, increasing from the sixth percentile to the 16th percentile from pre- to post-intervention (Table 2).

**Figure 2:**
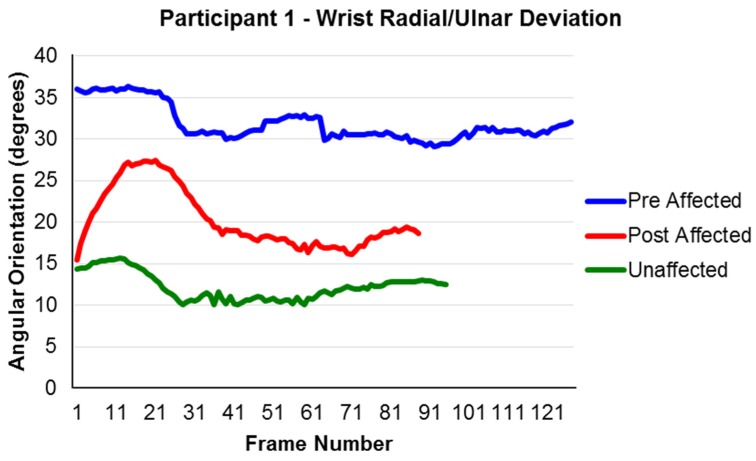
The Right Extended Forward Reach; (+) ulnar deviation and (−) radial deviation for Participant 1. Note: Frame number is a representation of time. The interval between frames was 1/60^th^ of a second. Participant 1 took longer to perform the reach prior to the intervention compared to after the intervention.

**Figure 3:**
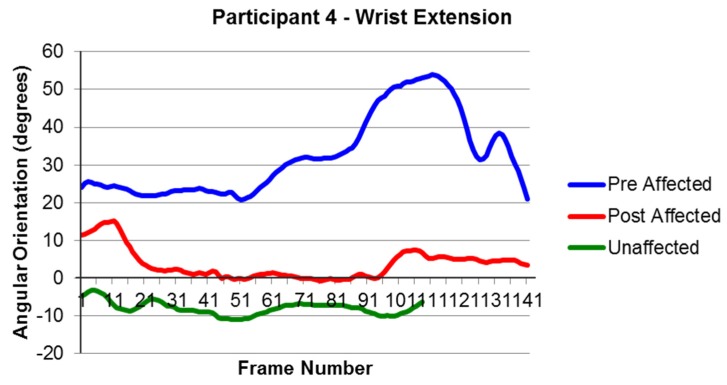
The Side Extended Reach; (+) flexion and (−) extension of Participant 4. Note: Frame number is a representation of time. The interval between frames was 1/60th of a second. The affected arm of Participant 4 took longer to perform the reach compared to the unaffected arm.

Data were able to be collected for the Modified Functional Reach Test both before and after the intervention [22,23]. For the data analysis, it was hypothesized that movement improvements were made if the movements progressed closer to similar movements of the unaffected arm. Changes were observed for joint movements; however, they varied across participants. For example, Participant 1 showed improvements in her radial/ulnar deviation during forward extended reach by displaying greater movement toward a more neutral position and closer to the motion of her unaffected side (Figure 2). Participant 4 showed an increase in wrist extension during the left side extended reach (Figure 3). As with Participant 1, his movements were both closer to a neutral position and closer to the motions of his unaffected side.

### 2.2. Level of Intrinsic Motivation during Training

**Figure 4:**
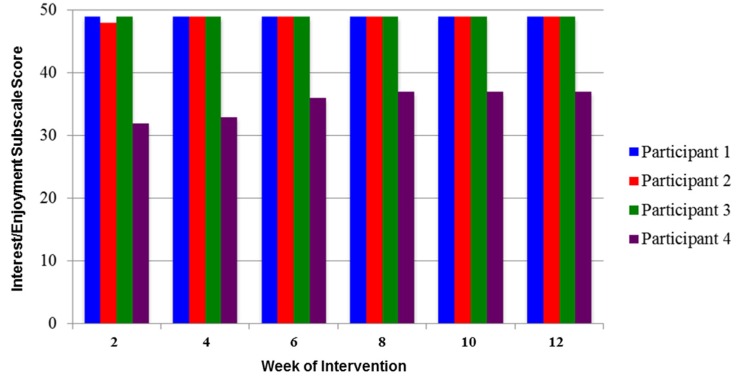
The score for the interest/enjoyment subscale of the Intrinsic Motivation Inventory (maximum score = 49).

The participants expressed high intrinsic motivation throughout the intervention. This was demonstrated by their average rating of 46 out of 49 possible points on the interest/enjoyment subscale of the Intrinsic Motivation Inventory (IMI) over the 12-week intervention (Figure 4).

A high level of motivation was also noted in the comments made by the participants. Participant 1 came to every session knowing what score she was aiming to beat on her basketball shot game. Participant comments included: “I really like playing, when do I get to do these at home?”, “I wish my teacher had this, so I could play it there.” (Participant 2), “I want to play this all day!” (Participant 3). “I remember when I was really bad at this game…like two weeks ago!” and “When can I play this at home, mom?” (Participant 4).

## 3. Discussion

The purposes of this investigation were to: (1) test the feasibility of delivering our upper extremity motor training intervention to children with cerebral palsy; and (2) determine the level of intrinsic motivation during intervention participation. There were five major limitations associated with the investigation. First, the purpose of the study was to determine the feasibility of the intervention. It was not to thoroughly investigate the changes that occurred as a consequence of it. Hence, only limited amounts of pre- and post-assessment data were presented to demonstrate feasibility. Larger, more controlled studies can demonstrate the potential effect of the intervention. Second, participants were on the higher functioning spectrum of our inclusion criteria. Their high functioning and involvement in multiple activities may have led to a ceiling effect for our selected assessments. While our goal was to confirm that we could collect the measures both pre- and post-intervention, care must be taken in selecting assessments that can match the abilities of the participants in future investigations. Further, we do not know how well participants that were more or less impaired would respond to the intervention. Since 91%–97% of children play videogames, it is likely that they could become engaged in the intervention [24,25]. Third, the Kinect sensor and FAAST software were unable to monitor movements of the hand and fingers where three of the participant had difficulties. It is possible that newer iterations of the Kinect may monitor hand movement, but that has not been tested. Fourth, we experienced periodic technical difficulties, which at times interrupted continuous play. The problems were solved, and play continued; yet, it is important to be aware that technical difficulties are possible. Finally, it should be noted that the IMI has been shown to be a valid and reliable instrument, including the interest and enjoyment subscale. However, it has not been previously administered with children with CP. The results should be considered carefully for this reason, as well as the small cohort.

The intervention feasibility was deemed successful based on the assessment criteria. Participants completed the 12-week intervention in both the laboratory and the home. Twenty-six free online videogames were used in conjunction with the Kinect motion sensor and the FAAST software to facilitate the intervention. Game scores continued to increase over the course of the intervention. Further, high numbers of repetitions were recorded for all participants during the 40 min of game play per session (average ~870). The high number of repetitions was greater than our other study with persons with stroke, where 250 repetitions were achieve during 20 min of game play [11]. These high repetitions enable current rehabilitation motor learning theory [26]. High meaningful repetitions are important in achieving brain remodeling (neuroplasticity) where new areas of the brain take on new functions to make up for areas that have experienced damage.

Feasibility was also successful in our ability to collect assessment data prior to and following the intervention. Three different levels of assessments were made, including impairment (AROM), motor performance (BOT-2) and function (Functional Reach Test). It was noted that a variety of assessments should be used to account for the high degree of variability among the participants.

The level of intrinsic motivation was high based on the scores from the interest/enjoyment subscale of the IMI [27]. High motivation was also supported by the comments made by the participants throughout the investigation. Our prior work with only a few videogames indicated that a child with CP quickly lost interest in playing videogames when the games were no longer a challenge and new games were not available [28]. It seems reasonable to assume that our ability to select games based on the child’s interest and to change the games when interest was waning (Table 1) had much to do with the high level of motivation throughout the 12-week intervention.

The current investigation adds to the body of knowledge from one major perspective. Free online videogames can be used in conjunction with the Kinect motion sensor (~$100) and the free FAAST software to create a highly motivating upper extremity motor intervention for children with CP . The use of free videogames is extremely novel and innovative. There is an endless supply of free videogames on the Internet covering any topic of interest. The videogames allow for matching the individual participant’s interests with specific games. The Kinect is able to monitor the participant’s movement and to feed the data to the FAAST software where individualized movement needs can be continuously challenged to elicit improvement.

The clinical implications of this study are that this tool can be used by therapist to motivate clients to obtain a large amount of challenging repetitions in the short amount of time allowed for therapy sessions. Future investigations should test the methods with additional participants and include a control group.

## 4. Materials and Methods

### 4.1. Participants

The current investigation recruited four participants with spastic hemiplegia CP (Table 4). All participants were actively involved in age-appropriate activities. Participants 1, 2 and 4 displayed impairments in their wrists on the affected side, while Participant 3 had impairments in the right shoulder. Informed consent was obtained from participant’s parents. All participants were identified as Level 1 of the Gross Motor Function Classification System (GMFCS) due to their ability to perform functions like running and jumping with impaired balance, speed and coordination. The GMFCS is a 5-level classification system that describes the gross motor function of children and youth with cerebral palsy on the basis of their self-initiated movement with particular emphasis on sitting, walking and wheeled mobility. Distinctions between levels are based on functional abilities, the need for assistive technology, including hand-held mobility devices (walkers, crutches or canes) or wheeled mobility and, to a much lesser extent, quality of movement [29].

All participants were identified as Level II of the Manual Ability Classification System (MACS) due to their ability to handle some object with reduced quality and the use of alternative methods for performing some tasks. The MACS was developed to classify how children with cerebral palsy (CP) use their hands when handling objects in daily activities. The five-level classification system is designed to reflect the child’s typical manual performance, not the child’s maximal capacity [30]. The Institutional Review Board at Washington University School of Medicine approved the study protocol. Participants continued pre-existing therapy and activities during participation.

### 4.2. Intervention

**Figure 5:**
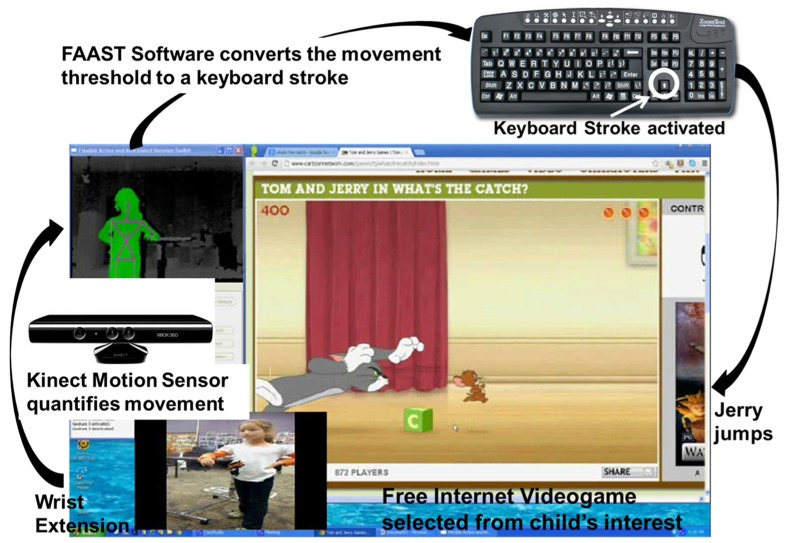
Movement therapy using free videogames, Kinect and FAAST software. The participant performs the motion. The Kinect converts segment/joint motion to XYZ coordinates. FAAST software identifies movement threshold and activates keyboard stroke. Jerry jumps.

The UE VR training system consisted of free Internet videogames, a Microsoft Kinect sensor [20] , the FAAST software, a computer and a 81 cm monitor (Figure 5). The Microsoft Kinect sensor was used to quantify the participants’ motion while playing the videogame and to send position data (X, Y, Z coordinates of body segments) to the FAAST software. The FAAST software: (1) monitored the body segment coordinates; (2) identified when a therapist-specified movement threshold was achieved; and then (3) activated a keyboard stroke/mouse movement. The keyboard stroke/mouse movement was that which was required to play the videogame. Hence, the Kinect streamed movement data of the participant to the FAAST software. The FAAST software monitored the joint/segment movement selected by the therapist (e.g., wrist extension) waiting for the movement threshold (e.g., 20° of extension), also chosen by the therapist. When the movement threshold was achieved, the FAAST software sent a keystroke signal (e.g., upper arrow key) to the videogame for game play (Figure 5).

Intervention sessions were aimed at obtaining high joint repetitions through single and combination joint movements. Sessions occurred three times per week (1 h) for 12 weeks. Each session consisted of the child performing five minutes of supervised UE stretching to warm-up. Next, the child played four different games while standing (each ~10 min), involving different UE movements for each game. Participants were given short rest breaks, between games as needed.

Joints undergoing movement training in the UE were based on each individual’s abilities derived from the child’s available active range of motion assessed during the pre-intervention assessment session. During each training session, the entire UE was engaged in game play. UE movements targeted during a session included shoulder abduction, shoulder flexion/extension, shoulder internal/external rotation, elbow flexion/extension and wrist flexion/extension, as needed. Increases in movement thresholds occurred individually as each child’s active range of motion increased to continually increase challenge.

Games played during the sessions were based on the interests each child expressed during a pre-intervention interview and through continued input throughout the training. Games varied throughout the intervention based on the child’s desires. While the games varied, targeted body areas remained constant. Each session provided a choice to the participant while targeting specific UE movements. One example included a child playing “Run Jerry Run” using right wrist extension to cause the mouse to jump over objects [31].

Training sessions progressed in a stepwise manner from completion in the laboratory to completion in the child’s home. Participants were provided with necessary hardware and software to complete sessions in the home. Experienced research assistants trained the parents to conduct the in-home sessions during the first 3 weeks. Training included: introduction to the project, explanation of the equipment setup and protocol instruction (total time ~2 h). Research assistants handled troubleshooting of any problems with hardware and software throughout the intervention.

### 4.3. Feasibility of Intervention Delivery

The feasibility of delivering the videogame motor training intervention was determined using four different methods. The first was whether the entire 12-week intervention (3 × /week) could be completed by the participant in both the laboratory and the home. This included the videogame motor training in the laboratory and home and the collection of assessment data to determine if any changes occurred as a result of the intervention. It also included instructing the parents and the child how to perform the training at home. The evaluation was performed by recording the motion data from the Kinect sensor in MATLAB (running in the background) during game play and the recorded documentation from the FAAST software output. The second method was quantifying the number of repetitions that typically occurred during a single training session. Repetitions recorded from MATLAB data were counted for each participant for each day and averaged across the intervention. The third method was monitoring the progression of game play over the course of the 12-week intervention. This was quantified by recording the high score of a single game over the intervention.

The final method was collecting three different types of assessments prior to the start and immediately after the completion of the training regimen to quantify any effects that could come about from the training program. All assessments were performed by a single experienced clinician with prior training in all measures.

The first was the child’s AROM which is an assessment designed to evaluate an individual’s active movement in different directions. The AROM measurements followed standard procedures using a goniometer. Measurements were taken for shoulder flexion, shoulder abduction/adduction, elbow flexion/extension and wrist flexion/extension. The use of goniometers is accepted as a valid clinical tool for collecting AROM [32]. It is desirable to have the same person complete the assessment on all of the participants. The AROM assessment prior to the start of the intervention was also used to determine targeted training movements for the intervention and set parameters for the degree of body movement thresholds during game play.

The second assessment type was the manual coordination subtest of the Bruininks–Oseretsky Test of Motor Proficiency (BOT-2). The BOT-2 is a standardized norm-referenced measure of fine and gross motor skills of children and youth, 4–21 years of age [33]. It is intended to be a discriminative and evaluative measure to characterize motor performance, specifically in the areas of fine manual control, manual coordination, body coordination and strength and agility. The manual coordination subtest quantifies the child’s ability to demonstrate skills, such as catching, throwing and dribbling a tennis ball with one or both hands.

The third assessment type was the Modified UE Functional Targeting Reach Test [22,23], used to evaluate UE motor control. This assessment utilized an 8-camera video motion capture system to detect children’s movement based on reflective markers placed on their UE. During this assessment, the child completed three reaches in the sagittal and coronal planes with each UE at an “easy” and “extended” distance [22]. Data from the reaching tasks were used to evaluate joint angles during reach. Joints examined included trunk (flexion/extension, lateral flexion, axial rotation), shoulder (abduction, elevation, internal/external rotation), elbow (flexion/extension, pronation/supination) and wrist (flexion/extension, ulnar/radial deviation) [11,22]. Angles were defined in relation to the more proximal body segment.

### 4.4. Level of Intrinsic Motivation during Training

The level of intrinsic motivation during training was monitored biweekly throughout the intervention using the interest/enjoyment subscale of the IMI. This subscale has been shown to reflect the overall level of intrinsic motivation an individual experiences when engaged in an activity [34]. Based on the developer’s guidelines, a total score from the seven questions was calculated. From a qualitative perspective, all verbal comments relative to the training made by the participant during the intervention were recorded in a SOAP (subjective, objective, assessment and plan) note.

## 5. Conclusions

This investigation determined the feasibility of delivering a videogame motor training intervention to four children with CP, as well as their level of motivation during play. The intervention used a free Internet videogame in conjunction with the Kinect motion sensor and the FAAST software. Results indicated that the intervention could be successfully delivered in the laboratory and the home, and pre- and post-assessments were possible. Results also indicated a high level of motivation among the small number of participants. Future work should include results from additional participants and from a control group in a randomized controlled trial to establish efficacy.

## Figures and Tables

**Table 1:** List and description of the 26 games played by the children during the intervention.

Game Name	Game Genre	Sub-genre	Goal of Game	Movement	Particpant
Refriger-Raiders (Jerry)	Cartoon	Object Collection	Move to cheese and pick it up, drop it to nibbles. Avoid getting hit by pool balls.	Wrist Extension	1, 2
Refriger-Raiders (Tom)	Cartoon	Throwing	Throw balloons to hit the target as Jerry passes through it	Wrist Extension	2
What's the Catch (Jerry)	Cartoon	Chase	Help Jerry reach his mouse hole before Tom catches him while avoiding objects	Shoulder Flexion; Wrist Extension; Shoulder Abduction	1, 2, 3, 4
Robot Unicorn Attack	Cartoon	Jumping	Jump to catch the ferries and stay on the platform. Avoid stars.	Shoulder Abduction; Elbow Extension	4
Fruit Ninja	Cartoon	Slicing Fruit	Using of sword to slice through fruit that fly up into screen	Elbow Flexion, Shoulder Flexion	1, 2
Tower-Inator	Cartoon	Sling Shot	Sling shot of bowling balls at structures to knock them over	Combination Reaching Movement (Shoulder circumduction)	1
Angry Birds	Cartoon	Sling Shot	Sling Shot of pigs at structures to knock them over	Combination Reaching Movement (Shoulder circumduction)	1
GrumbleGum	Cartoon	Object Collection	Take character along path in order to collect items	Shoulder Flexion	3
Star Wars: Jedi vs. Jedi	Cartoon	Fighting	Jedi fight against computer Jedi	Shoulder Fexion; Shoulder External Rotation	3
Shotgun vs. Zombie	Cartoon	Fighting	Character fighting and shooting zombies	Shoulder Flexion; Shoulder Abduction	4
Lateral Collateral 2	Sports	Football	Get the ball to the endzone without being tackled. Pass the ball back and forth to teamates and move up and down the field.	Bilateral Elbow Flexion; Shoulder Abduction	2, 3, 4
Highway Madness	Sports	Car Racing	Drive down the road avoiding traffic and collecting bonuses to complete the mission.	Shoulder Flexion, Shoulder External Rotation	3
Penalty Shootout	Sports	Soccer	Aim and shoot the ball into the net. Avoid the goalie.	Combination Reaching Movement (Shoulder circumduction)	3
Hoops Mania	Sports	Basketball	Make as many baskets in a row as possible.	Elbow Flexion; Shoulder Abduction; Shoulder External Rotation; Shoulder Internal Rotation	1, 3, 4
Air Hockey	Sport	Hockey	Move hand around to defend goal and shoot puck	Combination Reaching Movement (Shoulder circumduction)	1, 3
Marathon Runner	Sport	Running	Jump over obstacles while running	Elbow Flexion	1
Upstream Kayaking	Sport	Kyaking	Direct kayaker around obstacle	Elbow Flexion	1
G-Switch	Sport	Running	Move character while running to avoid obstacles	Elbow Flexion	1
Basket Shot	Sport	Basketball	Make as many baskets in a row as possible.	Elbow Flexion, Wrist extension	1, 2, 4
Harry Potter Quiddithch	Sport	Quidditch	Blocking computer player from scoring in goals	Combination Reaching Movement (Shoulder circumduction)	1, 3
Cyclomaniacs	Sport	Bicycling	Guiding bike along path	Shoulder Abduction; Shoulder Flexion	3
Spiderman Racing	Sport	Bicycling	Guiding bike along path	Shoulder Abdcution; Shoulder External Rotation	4
Ulitmate Baseball	Sport	Baseball	Batting within baseball game	Wrist Extension	4
1 on 1 Soccer	Sport	Soccer	Playing Soccer against computer person	Shoulder Abduction; Wrist Extension	4
Guitar Geek	Music	Guitar	Hit the notes at the right time to play the guitar	shoulder external rotation; shoulder flexion	1
Music Catch 2	Music	Object Collection	Move select hand around the screen in order to catch the falling music notes	Combination Reaching Movement (Shoulder circumduction)	1

**Table 2:** The pre- and post-intervention upper extremity joint active range of motion results for the participants.

Participant (#)	Shoulder Flexion		Shoulder Extension		Shoulder Abduction		Shoulder Internal Rotation		Shoulder External Rotation		Elbow Flexion		Elbow Extension		Wrist Flexion		Wrist Extension	
	Pre	Post	Pre	Post	Pre	Post	Pre	Post	Pre	Post	Pre	Post	Pre	Post	Pre	Post	Pre	Post
1	155	150	50	50	155	146	75	80	45	50	140	145	0	0	75	67	0	7
2	160	150	60	52	150	158	75	68	55	50	140	140	0	0	30	25	0	5
3	127	140	50	33	134	145	54	72	57	70	145	136	0	0	70	69	0	9
4	147	160	55	47	140	144	40	53	67	88	160	152	0	0	3	50	0	35

Note: Measurements in degrees.

**Table 3:** The pre- and post-intervention BOT-2 standard scores for Participants 3 and 4.

Participant (#)	Manual Dexterity		Upper-Limb Coordination		Manual Coordination		% Rank	
	Pre	Post	Pre	Post	Pre	Post	Pre	Post
3	2	3	7	7	28	29	1%	2%
4	6	7	11	15	34	40	6%	16%

**Table 4:** Demographic information about participants recruited for investigation.

Participant (#)	Age (y)	Gender	Affected Side	* GMFCS Level	** MACS Level
1	17	Female	Right	I	II
2	8	Female	Right	I	II
3	10	Male	Right	I	II
4	9	Male	Left	I	II

Note: * Level 1 of the Gross Motor Function Classification System (GMFCS) due to their ability to perform functions like running and jumping with impaired balance, speed and coordination; ** Level II of the Manual Abilities Classification System (MACS) due to their ability to handle some object with reduced quality and use of alternative methods of performing some tasks.

## Abbreviations

The following abbreviations are used in this manuscript:

AROM: active range of motion
BOT-2: Bruininks-Oseretsky Test of Motor Proficiency
CP: cerebral palsy
FAAST: Flexible Action and Articulated Skeleton Toolkit software
IMI: Intrinsic Motivation Inventory
ROM: range of motion
UE: upper extremity
